# Supplementary figures and images for: Mitochondrial VDAC1 Silencing in Urethane-Induced Lung Cancer Inhibits Tumor Growth and Alters Cancer Oncogenic Properties
Source: Cancers (Basel). 2024 Aug 26;16(17):2970. doi: 10.3390/cancers16172970 (PMC11393979; doi:10.3390/cancers16172970)

## Figure 1B

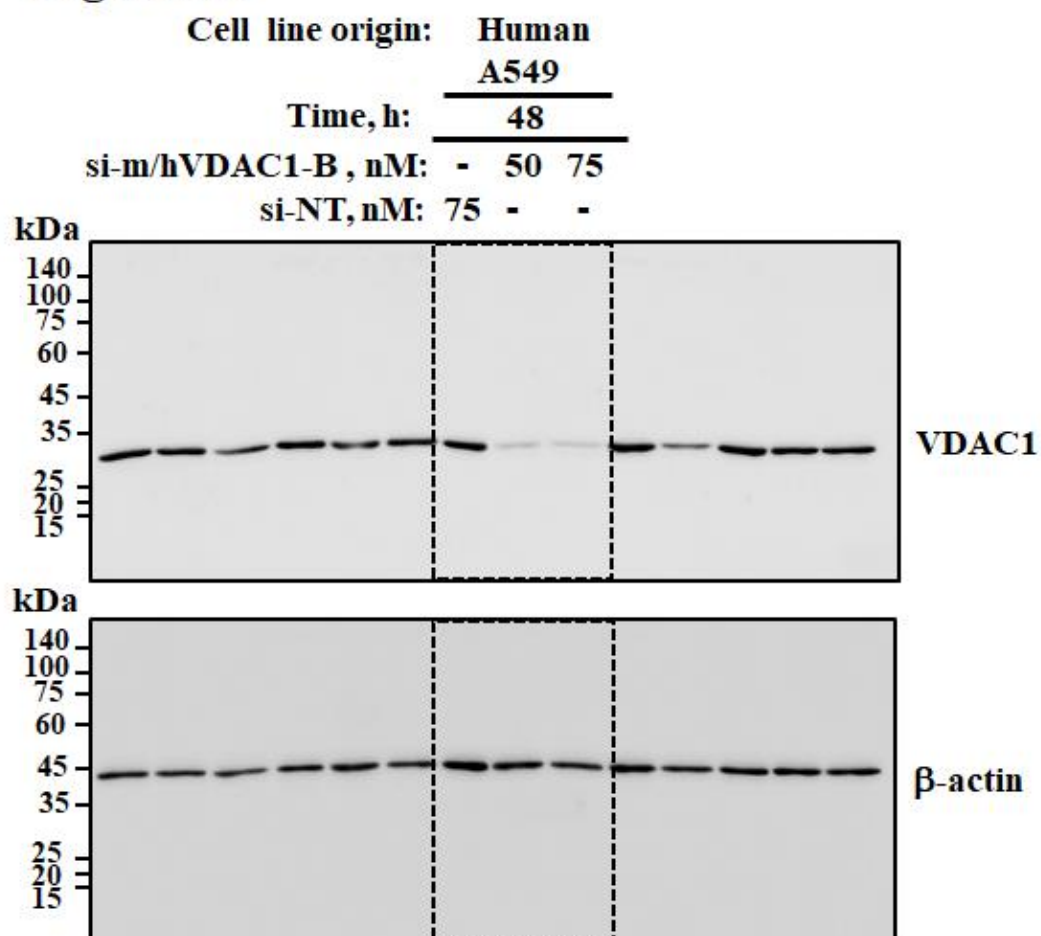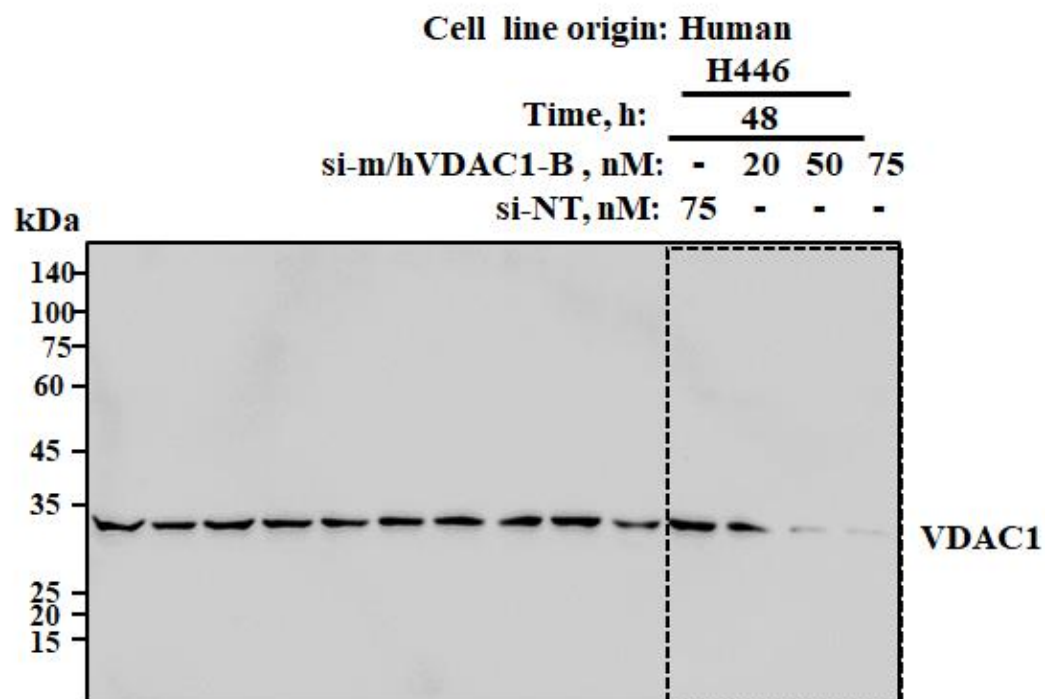

## Figure 1B

Cell line origin:

2LL

Time, h: 48 72 48 48 72 72

si-NT, nM: 75 75 - - - -

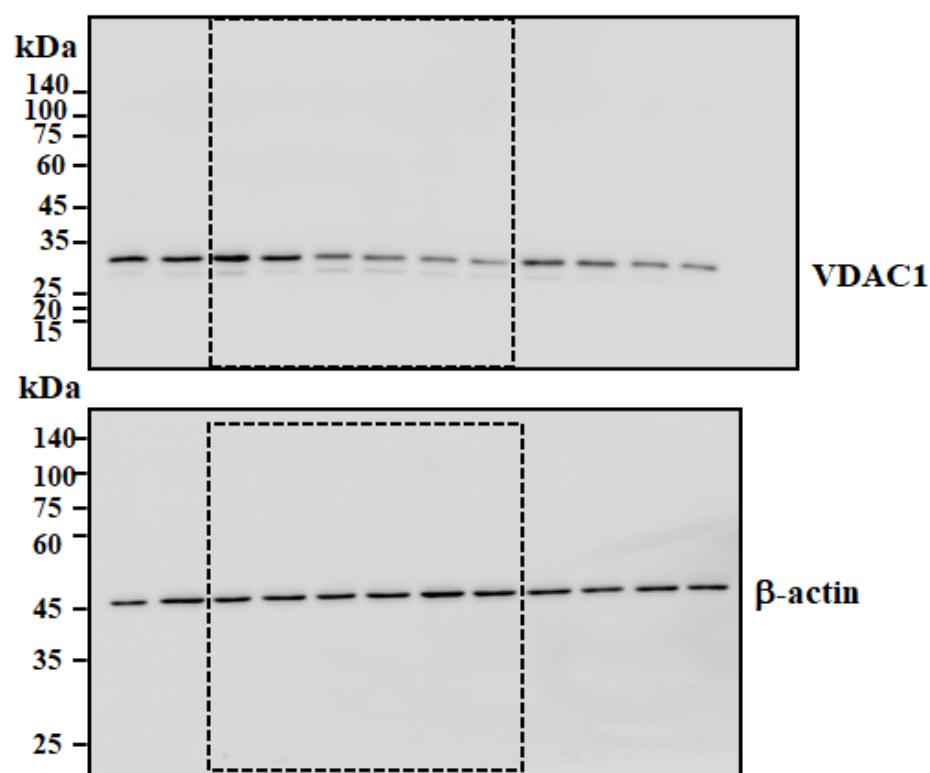

## Figure 7A

si-m/hVDAC1-B, nM: - 20 50 75

si-NT, nM: 75 - - -

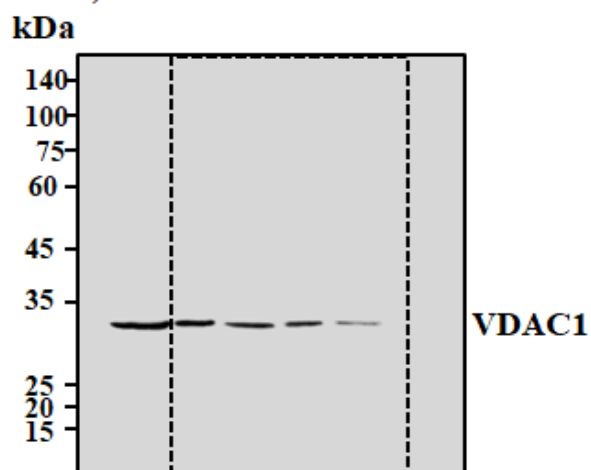

Supplement: Supplementary file 1 [file cancers-16-02970-s001.zip › cancers-3101111-supplementary-File S1.pdf]
